# Health-related quality of life of adolescents conservatively treated for idiopathic scoliosis in Korea: a cross-sectional study

**DOI:** 10.1186/s13013-016-0071-1

**Published:** 2016-03-31

**Authors:** Hyejung Lee, Jihea Choi, Jin-Ho Hwang, Jung Hyun Park

**Affiliations:** College of Nursing, Yonsei University, 250 Seongsan-ro, Seodaemun-gu, 03722 Seoul Korea; Department of Nursing, Yonsei University Wonju College of Medicine, 20 Ilsan-ro, Wonju-si, 26426 Gangwon-do Korea; Department of Orthopaedic Surgery, Severance Children’s Hospital, Division of Pediatric Orthopedics, Yonsei University College of Medicine, 50-1 Yonsei-ro, Seodaemun-gu, 03722 Seoul Korea; Department of Rehabilitation Medicine, Gangnam Severance Hospital, Rehabilitation Institute of Neuromuscular Disease, Yonsei University College of Medicine, 211 Eonju-ro, Gangnam-gu, 06273 Seoul Korea

**Keywords:** Adolescent idiopathic scoliosis, Health-related quality of life, Scoliosis research society, Conservative treatment, Cross-sectional study

## Abstract

**Background:**

Young adolescents with scoliosis are more likely than adults to experience psychological distress affecting health-related quality of life (HRQoL). Adolescence is a sensitive period of psychological development, and thus physical deformity from scoliosis can negatively affect body image and appearance of adolescents. The present study evaluated HRQoL in young Korean adolescents with idiopathic scoliosis and identified related factors.

**Methods:**

One hundred and ten adolescents with idiopathic scoliosis were recruited from two tertiary hospital outpatient clinics over one year. HRQoL was measured using the Korean version of the Scoliosis Research Society 22 revision (SRS-22r) questionnaire. In addition, participant medical records were reviewed to collect data on severity of scoliosis, type of treatment and age at which they were first diagnosed with the disease.

**Results:**

The mean age of the participants was 14.2 years and 48.2 % were first diagnosed at 9–12 years. Most participants (61.8 %) were under observation to follow up the curvature progression and 20.9 % received regular physiotherapy. Almost half the participants (47.3 %) had mild (10°–25°), 41.8 % moderate (25°–40°), and 10.9 % severe (>40°) scoliosis. The total score of the SRS-22r differed significantly between the groups of age at diagnosis (*p* = 0.033) and type of treatment (*p* = 0.025). Self-image, a sub-domain of the SRS 22r, was significantly lower in the severe curve deformity group than in the other groups (*p* = 0.031).

**Conclusions:**

An earlier age of scoliosis diagnosis and conservative treatment were related to higher HRQoL scores of Korean adolescents with scoliosis. Although the overall HRQoL did not significantly differ by severity of disease, self-image was significantly decreased in adolescents with severe spinal deformity. HRQoL of adolescent patients with idiopathic scoliosis can be affected by several factors that medical staff needs to consider in order to produce the best and most effective treatment outcomes.

## Background

Scoliosis is a complex deformity of the spine involving a three-dimensional deviation of the spinal axis, and is diagnosed by the spinal curvature exceeding 10° on an anterior-posterior X-ray image [1]. Adolescent idiopathic scoliosis (AIS) is the most common type of scoliosis, accounting for more than 80 % of scoliosis cases [2]. The prevalence rate of AIS in Korea is 0.19–3.9 % [3], similar to the 2–4 % in the United States [4] and 0.87 % in Japan [5].

Treatment for scoliosis consists of either surgery or conservative interventions, and is usually decided on the basis of the initial Cobb angle and progression of spinal deformity. In general, conservative treatment, consisting of periodic follow-ups, exercise (physiotherapy), and brace application, is recommended for mild (Cobb angle <25°) or moderate scoliosis (Cobb angle of 25°–40°). Surgical correction and stabilization of the spine is considered for severe scoliosis (Cobb angle >45°) [1]. Unless adequate treatment is provided in a timely manner, AIS can lead to increasing physical, psychological, and social problems such as limited physical activities, musculoskeletal pain, poor body image, self-depreciation, maladjustment at school, and difficulty in peer relationships [6, 7]. These consequences of scoliosis may also affect quality of life in adolescents with scoliosis [8].

Surgical treatment for scoliosis significantly increased self-image, self-esteem, and life-satisfaction of adolescent patients [9–11]. However, conservative treatments were not shown to improve the mental health of adolescents in one study [12], while in another study of female adolescents, mental health did not deteriorate during the course of conservative treatment [9]. Thus, the psychosocial aspects of adolescents with scoliosis seem to be affected by type of treatment received.

Health-related quality of life (HRQoL) tools have been used to measure the effect of treatment in scoliosis patients. The HRQoL of conservatively treated AIS patients did not differ between those that were braced and those under observation-alone management [13]. Additionally, HRQoL was shown to differ according to type of brace used [14]. Time spent in the brace is shown to be related to body image, which decreased with time spent in the brace [15]. However, HRQoL measured after 25 years of treatment has been shown to be similar to the norm regardless of the type of treatment received [15]. Thus, the effect of scoliosis treatment on HRQoL is influenced by time [16]. Magnitude of curvature is an established factor influencing the HRQoL of adolescents and adults [17]; the larger the magnitude, the poorer the HRQoL [17]. However, Rainoldi et al. [18] reported a very low correlation with deformity severity and small differences in HRQoL between small and large curve magnitude. In addition to these disease-severity factors, gender also affects HRQoL, with a higher HRQoL shown in boys, and lower self-image in girls [13, 17].

Adolescence is a critical period of psychological development. The physical deformity caused by scoliosis may lead to social and psychological pressure on developing adolescents [12]. Considering that scoliosis is more prevalent in female adolescents, and that girls might be more concerned about physical appearance than boys, a better understanding of disease-related psychological aspects is needed to inform clinical practice [19]. Psychological stress can be correlated with lack of compliance to brace treatment in females with scoliosis [8, 20].

The Scoliosis Research Society 22 revision (SRS-22r) questionnaire has been widely used as a reliable and valid instrument to measure HRQoL internationally [11, 21] since it was developed to assess patients’ perception of their condition in addition to medical treatment effects [22–24]. The Korean version of the SRS-22r was developed and validated after cross-cultural adaptation and translation [24]. However, few studies have employed the SRS-22r to investigate HRQoL in Korean adolescents with scoliosis. Thus, the present study described and compared HRQoL of adolescents with idiopathic scoliosis by disease-related characteristics such as age of diagnosis, type of treatment, severity of scoliosis, as well as demographic characteristics.

## Methods

One hundred and ten adolescents with idiopathic scoliosis were recruited from an orthopedic and rehabilitation outpatient clinic in two tertiary hospitals located in Seoul, Korea. The inclusion criteria for the study were as follows: primary diagnosis of AIS determined by clinicians, aged 10–19 years, and agreement to participate by participants and their legal guardians. The exclusion criteria consisted of having any other diagnosable musculoskeletal disease except scoliosis, and cognitive impairment.

Prior to the initiation of the study, approvals from two hospitals (K hospital: IRB No. 3-2010-0172, S hospital: IRB No. 4-2011-0682) were obtained. Primary physicians or a research assistant verbally explained the purpose and intention of the study to potential participants and invited their participation. Adolescents and their legal guardians who agreed to participate signed the informed consent, and then completed the questionnaires. All participants completing the survey were monetarily remunerated.

The self-report questionnaires included questions about personal characteristics such as age, gender, and age at diagnosis, in addition to the SRS-22r questionnaire. The SRS-22r contains 22 questions in five domains: function (5 items), pain (5 items), self-image (5 items), mental health (5 items), and satisfaction with management (2 items). Each item contains a 5-level Likert scale ranging from worst (1 point) to best (5 point); results are expressed as the mean score of each domain, and total score of the scale. A higher total score indicates a higher level of quality of life. Reliability of the scale (using Cronbach’s α) was 0.84 in this study.

Participant medical records were also reviewed to collect disease-related data such as the present Cobb angle, and the type of treatment they were receiving. Cobb angle was employed to determine severity of disease, which was classified into three groups: mild (10°–25°), moderate (25°–40°), and severe (>40°). Type of treatment was categorized as: observation, brace, physiotherapy, and brace with physiotherapy. Observation referred to regular clinical check-up for curvature progression without any active treatment. Physiotherapy included bi-weekly participation in the exercise program provided in the hospital outpatient clinic and practice with home-based exercise.

Data were analyzed using PASW Windows version 20.0. *P* values < 0.05 were considered statistically significant. Descriptive statistics, ANOVA, Kruskal-Wallis, and Mann–Whitney U tests were used to compare HRQoL, with non-parametric analyses used when data was not normally distributed and group participant numbers were uneven.

## Results

Most participants (80.9 %) were female, the mean age was 14.2 (SD 2.17) years, and the mean age at diagnosis with AIS was 12.5 (SD 1.82) years. Almost half the participants (48.2 %) were diagnosed at the age of 9–12 years, while the other half (44.5 %) were diagnosed between 13–15 years. The majority of participants (61.8 %) were under observation with regular check-ups for spinal curvature changes, 2.7 % received brace application, and 20.9 % received physiotherapy (Table 1). Among the 110 participants, 52 (47.3 %) were categorized as having mild, 46 (41.8 %) as having moderate, and 12 (10.9 %) as having severe scoliosis.

**Table 1:** Characteristics of patients (*N* = 110)

Characteristics	N (%) or Mean (SD)/range
Gender	
Male	21 (19.1)
Female	89 (80.9)
Age, years	14.2 (2.17) 10-19
Age at diagnosed, years	12.5 (1.82) 9-16
9-12 (School age)	53 (48.2)
13-15 (Junior high school age)	49 (44.5)
16 (High school age)	8 (7.3)
Duration of treatment received, months	21.11 (17.41) 6-72
Type of management	
Under observation	68 (61.8)
Brace therapy	3 (2.7)
Physiotherapy	23 (20.9)
Brace & Physiotherapy	16 (14.5)
Type of curve	
Thoracic	31 (28.2)
Thoracolumbar	7 (6.4)
Lumbar	21 (19.1)
Double major	51 (46.4)
Curve severity at present	
Mild (10° < Cobb’s angle ≤ 25°)	52 (47.3)
Moderate (25° < Cobb’s angle ≤ 40°)	46 (41.8)
Severe (40° < Cobb’s angle)	12 (10.9)

The mean total score of the SRS-22r was not different according to gender and curve severity. However, total SRS-22r scores differed between age group at diagnosis with AIS (*p* = 0.033), and type of treatment (*p* = 0.025) (Table 2). Adolescents diagnosed with AIS at the age of 9–12 years showed significantly higher scores in the SRS-22r compared to adolescents diagnosed at the later age (13–15 years). Lowest SRS-22r scores were found in adolescents receiving physiotherapy only. The median score of the SRS-22r was not significantly different between the three severity groups (*p* = 0.137), but the median score of the self-image sub-scale differed significantly between groups (*p* = 0.031), with the severe group having a lower score than the other groups (Table 3).

**Table 2:** Health-related quality of life among adolescents with idiopathic scoliosis by characteristics (*N* = 110)

Characteristics	HRQoL (SRS-22r)	
	Mean (SD) or ɤ	*p* (Post-hoc)
Gender		
Male	4.18 (0.46)	0.061
Female	4.21 (0.35)	
Age at diagnosed, years		
9-12 (School age) a	4.30 (0.29)	0.033 (a > b)
13-15 (Junior high school age) b	4.12 (0.43)	
16 (High school age)	4.15 (0.37)	
Type of management		
Under observation	4.28 (0.31)	0.025
Brace therapy	4.39 (0.33)	
Physiotherapy	4.03 (0.48)	
Brace & Physiotherapy	4.12 (0.35)	
Type of curve		
Thoracic	4.12 (0.40)	0.867
Thoracolumbar	4.28 (0.19)	
Lumbar	4.25 (0.35)	
Double major	4.19 (0.38)	
Curve severity		
Mild (10° < Cobb’s angle ≤ 25°)	4.30 (1.36)	0.137
Moderate (25° < Cobb’s angle ≤ 40°)	4.20 (1.45)	
Severe (40° < Cobb’s angle)	4.07 (2.05)	

Post-hoc: Scheffe

**Table 3:** HRQoL among adolescents with idiopathic scoliosis by severity of disease

Variable	Mild (*n* = 52)	Moderate (*n* = 46)	Severe (*n* = 12)	*p*
	Median (IQR^a^)			
HRQoL (SRS-22r)	4.30 (1.36)	4.20 (1.45)	4.07 (2.05)	0.137
Function/activity	5.00 (1.40)	4.80 (1.60)	4.50 (1.80)	0.053
Pain	4.60 (1.80)	4.60 (2.00)	5.00 (2.20)	0.692
Self-image/appearance	3.80 (2.20)	3.80 (3.00)	3.00 (2.80)	0.031
Mental health	4.20 (2.40)	4.00 (2.00)	4.10 (2.80)	0.414
Management satisfaction	4.00 (2.00)	4.00 (2.00)	4.00 (2.00)	0.782

^a^IQR: Inter-quartile range

## Discussion

The rapid physical growth in children just before puberty is a risk factor for idiopathic scoliosis. Today, earlier puberty seems to occur in many children in Korea, which in turn accelerates the increase of prevalence rate of idiopathic scoliosis. The present study investigated Korean adolescents who were conservatively treated for AIS to determine their quality of life and its related factors.

HRQoL of adolescents with idiopathic scoliosis, as measured by the SRS-22r, differed by age of diagnosis and type of treatment. Adolescents who were diagnosed earlier (9–12 years) reported higher HRQoL than adolescents who were diagnosed at a later age. This may be attributed to milder severity of the disease when diagnosed at an earlier age. For an early detection of scoliosis and prevention of surgical correction, school screening for scoliosis has been recommended in many countries despite its low predictive value [9]. Early detection of scoliosis helps to identify young children with milder curve deformity, and leads to conservative treatment with regular check-ups of spine curvature progression or physiotherapy. In Korea however, school screening for scoliosis has not been implemented. Instead, a self-reported questionnaire asking about back pain and posture problems is included in the regular school health examination scheduled upon school entry at ages 13 and 16 years. A chest X-ray examination for pulmonary tuberculosis, one item of the regular school health examination, is often used as a means for detecting AIS. Suspicion reagrding idiopathic scoliosis can often be noticed by a radiologist and referred for further examination [25]. Many adolescents in the current study might have received their diagnosis from the school health examination, although it was not asked how the scoliosis was detected in each participant. The American Academy of Pediatrics recommends scoliosis screening at ages 10, 12, 14, and 16 years [12], and the Scoliosis Research Society recommends annual scoliosis screening of all children aged 10–14 years [10]. Early and regular school screening for scoliosis is warranted in Korea to promote earlier diagnosis and to potentially prevent severe spinal deformity, which results in better HRQoL of adolescents affected, as indicated in this study.

A number of studies have reported that conservative treatment does not impact the quality of life of adolescents with scoliosis [26]. In contrast, our study showed that adolescents under observation or brace therapy showed higher HRQoL than adolescents receiving physiotherapy and who continued exercising at home. Whether our contrasting observation relates to ethnicity and sample differences may warrant further investigation.

In general, bracing is known to affect quality of life, which further depends on the type of brace employed [14, 17]. However, Danielsson and associates [27] reported that regardless of bracing, adolescents with less body asymmetry and similar curve size had higher quality of life, perhaps pointing to how adolescents perceive their body appearance being more important than the bracing itself. Further, non-braced adolescents showed less distortion in their body appearance than braced adolescents, therefore emphasizing the need for managing psychosocial aspects in AIS treatment.

Previous investigations have shown that HRQoL is inversely correlated with severity of spinal curvature in adolescents with idiopathic scoliosis [26], which improves when the spinal deformity is corrected by surgery [28]. In contrast, we found no difference in HRQoL by curve severity. This may be because most participants in this study had mild or moderate severity of spine curvature. However, scores of self-image differed significantly between severity groups in our study. Perception of self-image in the severe group (Cobb angle > 40°) was worse than in the mild or moderate group. This result is consistent with studies on conservatively treated patients [9, 25]. Self-image is one of the major factors affecting the development of friendships and the ability to adapt socially in adolescents [9]. Adolescent girls have a higher prevalence for idiopathic scoliosis than boys. Given that body dissatisfaction is more common among adolescent girls [7], each domain of quality of life should be assessed in adolescents with severe curve deformity with special consideration paid to gender.

A limitation of this study was the relatively small number of participants in each group of disease severity and treatment type making sub-group comparisons difficult; a future study recruiting a larger sample across a wider age range of adolescents is necessary. A prospective longitudinal study design would represent an improvement to aspects of retrospective analysis undertaken with our methods. Multiple regression analysis is required to examine the factors affecting HRQoL simultaneously. In addition, studies using a variety of reliable and valid instruments to measure HRQoL may be necessary to identify subtle differences in health-related outcomes in adolescents with progressive spinal curve disease.

## Conclusion

Young female adolescents with spinal deformity are more likely to experience psychological problems from a deformity of the spine. An earlier scoliosis diagnosis and undertaking conservative treatment are related to higher HRQoL. The self-image is worse in adolescents with severe curvature of the spine than in those with mild or moderate curvature. Regular school scoliosis screening is warranted to optimize the early detection of milder scoliosis in all children and adolescents. Screening only risk groups such as females during early puberty may be more cost effective than universal screening for scoliosis. Medical personnel need to routinely assess body image disturbances in adolescents with scoliosis during treatment in order to provide early psychological support when necessary, and to increase compliance with conservative treatment deemed essential for a successful outcome.
